# Atomic force microscopy-based topographical imaging of SARS-CoV-2 as part of a tripartite strategy for RNA virus characterization

**DOI:** 10.1186/s12967-025-07490-3

**Published:** 2025-12-09

**Authors:** Tanja Deckert-Gaudig, Xiaobin Yao, Erwan Darussalam, Franziska Hornung, Pablo Carravilla, Ziliang Zhao, Kourosh Rezaei, Christian Eggeling, Stefanie Deinhardt-Emmer, Volker Deckert

**Affiliations:** 1 Leibniz Institute of Photonic Technology, Jena, Germany; 2 Institute of Physical Chemistry, Friedrich-Schiller University Jena, Jena, Germany; 3 Institute of Medical Microbiology, Jena University Hospital, Jena, Germany; 4 Institute of Applied Optics and Biophysics, Friedrich-Schiller University Jena, Jena, Germany; 5 Department of Women’s and Children’s Health, Karolinska Institutet, Stockholm, Sweden; 6 Abbe Center of Photonics, Friedrich-Schiller University Jena, Jena, Germany

**Keywords:** Atomic force microscopy, RNA virus, SARS-CoV-2

## Abstract

**Background:**

The pre-selection of virus particles based on size and morphology is a crucial step toward rapid and reliable virus identification. Pre-selecting virus particles based on size and morphology represents a critical step toward rapid and reliable identification, which is particulary important in clinical settings when novel virus variants emerge. Although conventional fluorescence imaging enables visualization of specific viral structures via labeling, it does not allow for reliable differentiation of structurally similar particles.

**Methods:**

In this study, we present a combined imaging approach that integrates atomic force microscopy (AFM) and double-staining fluorescence microscopy to identify SARS-CoV-2 as a model RNA-virus from other sample constituents.

**Results:**

Initially, topographical imaging via AFM enables high-resolution visualization of individual virus particles, providing detailed information about particle morphology and height. Subsequently, dual fluorescence labeling of the RNA-containing core and the spike protein-rich surface allows for specific identification of intact viral structures. Correlation of fluorescence signals with AFM-derived height maps offers a comprehensive view of the particles’ morphological and molecular characteristics.

**Conclusion:**

This triple-correlation strategy enables the identification of intact SARS-CoV-2 particles and their clear distinction from similarly sized hollow particles, viral fragments, and staining artifacts. The height range of confirmed SARS-CoV-2 particles under the applied conditions was determined to be 60–100 nm. The presented correlative imaging approach is broadly applicable to other RNA viruses, offering a versatile tool for high-specificity virus detection.

**Supplementary Information:**

The online version contains supplementary material available at 10.1186/s12967-025-07490-3.

## Introduction

The COVID-19 pandemic revealed the need for complementary methods for a rapid and reliable virus characterization. This is in particular important for emerging viruses, especially at an early stage of virus outbreaks, when little morphological and genetic information about the pathogen is available. It is not only important for the understanding of virus-pathogenesis but also to preselect and identify virus from patient sample unambiguously without elaborate sample preparation.

In this respect, high resolution imaging techniques can contribute significantly. Among advanced microscopy techniques, (cryo-)electron microscopy (EM) and transmission electron microscopy (TEM) have been demonstrated as valuable tools to visualize the morphology of viruses with atomic resolution [1–5]. With these techniques, not only individual viruses can be studied but particles bound to infected host cell surfaces. Since such imaging techniques lack characterization of molecular structure and usually require sophisticated sample preparation protocols, novel approaches combine, for example, vibrational spectroscopy with scanning probe microscopy. This way, it is not only possible to characterize viruses chemically, but also to differentiate and classify them at the level of individual particles [6–10]. To perform a molecule-specific virus analysis in a targeted manner, using for example near-field optical methods, a preselection based on morphological characteristics is desirable to increase measurement turn-over rates. A previous study has shown that different viruses can be classified and distinguished from other sample components based on AFM topography, shape, and size alone through multivariate data analysis [11]. An important prerequisite for such approaches is a reliable sample preparation in which the viruses are isolated, inactivated and immobilized reproducibly to preserve their original morphology. In addition, the protein structure should also remain unchanged to facilitate a molecular structure characterization. The same applies to correlative studies, where different techniques are used on the same sample to characterize and increase the information content, e.g., for improved diagnosis.

Recent studies on severe acute respiratory syndrome coronavirus type 2 (SARS-CoV-2) clearly demonstrated that both sample preparation and environmental conditions are important parameters in AFM and EM measurements of viruses [2, 12–21]. SARS-CoV-2 is a single-stranded RNA virus belonging to the *coronaviridae* family. The capsid around the RNA containing core is enveloped by a lipid membrane which in turn is decorated with protruding spike proteins. The latter are highly flexible and susceptible to mechanical influences and may fall off during sample treatment, leaving gaps in the corona [22, 23]. This fact should be considered when purifying and handling SARS-CoV-2 for analysis. For example, the presence of the protein corona in TEM and AFM images suggests that chromatography is less harsh than ultracentrifugation for such sensitive samples [12]. 

The size ratio of the virus has been reported as follows: diameter ~ 100 nm, volume ~ 10^6^ nm^3^ and mass ~ 10^3^ MDa [13]. (Cryo-)EM studies have provided detailed reference information on the morphology and dimensions of this virus strain and values between 65 and 140 nm were reported [2, 12, 14–22, 24–26]. Despite theaccuracy of EM, these results already indicated that virus purification and fixation as well as the measurements conditions (e.g. negative staining, vacuum, cryogenic temperature) may affect the shape and dimensions of the viruses including deformations and the loss of the delicate protruding spikes. Furthermore, to obtain three-dimensional information from SARS-CoV-2, complex angle-dependent cryo-EM or tomography measurements of embedded sections with subsequent image reconstruction were necessary.

If the virus morphology, dimensions and mechanical properties are to be examined at ambient conditions in the dried state or under native liquid conditions, AFM is the most suitable method [11, 27–30]. One major advantage of AFM is that after virus isolation, the samples do not need any special further treatment but can be directly deposited on a substrate and examined. For our model virus, several AFM studies under different conditions are already available [23, 31–33]. The reported values of size vary between 40 and 140 nm and demonstrate how sample purification, inactivation method, substrate and imaging conditions affect the morphology and height of SARS-CoV-2, which is a generally common phenomenon for virus imaging with an AFM [27]. Consequently, the same protocol should be used for comparative studies.

In this context, it should be noted that virus sizes determined with an AFM may differ from those obtained with an EM. It is a commonly observed phenomenon when working with EM methods that morphology changes due to dehydration during fixation must be taken into account [34], except when working under cryogenic conditions. Therefore, the comparison of size data obtained with different imaging techniques should be treated cautiously [29–31]. 

Fluorescence microscopy can be considered as a gold standard to identify viral particles [35, 36]. Previous studies have shown the potential of fluorescent imaging of single SARS-CoV-2 particles, for example in high-throughput studies of host-virus interactions [37] to detect and identify viruses in clinical samples [38] or to study the binding of antiviral compounds [39]. While providing clear advantages, like labelling specificity, standard fluorescence microscopy also has some limitations. Generally, the resolution is diffraction limited to ca. 250 nm, well above the size of SARS-CoV-2. Moreover, common labelling strategies employ antibodies or fluorescent versions of viral proteins that only label one specific component, and cannot report on the overall virus structure, i.e., they cannot determine if a virion is fully assembled. Correlative imaging approaches have the potential to provide additional insights by detecting a combination of several viral particle features. For example, topography can be combined with fluorescence microscopy to measure viral particle size [40, 41]. 

In this contribution, chemically inactivated SARS-CoV-2 containing samples were firstly imaged with an AFM, to obtain topographic (specifically height) information. For the topgraphy-fluorescence microscopy correlation studies, the topography of virus particles with a stained RNA-containing core was imaged. It is notable that the staining process does not significantly change the height of the virus. Subsequently, these AFM images were compared with the corresponding confocal fluorescence microscopy images obtained from the excited dye in the stained core. Finally the spike protein-covered envelope was labeled with its specific antibody and fluorescence microscopy imaging was repeated for the attached dye. This labeling approach with dyes absorbing at different wavelengths was chosen to obtain double verification and a clear differentiation of intact viruses from detached RNA and spike protein fragments, incomplete and hollow viruses, and other sample components. In addition, the height range of the virions could be determined to 60–100 nm for immobilization on poly-L-lysine coated glass substrates and inactivation with paraformaldehyde (PFA).

Taken together, the presented correlative approach offers a robust and versatile framework for the early characterization of RNA viruses, including arboviruses, influenza viruses, and coronaviruses, which are among the most likely candidates to trigger future pandemics. By combining high-resolution topographical imaging with targeted molecular labeling, this method enables the rapid identification and differentiation of intact viral particles with high specificity, even in complex biological samples. Its adaptability to other virus types through the use of virus-specific antibodies makes it particularly valuable for deployment in early outbreak scenarios, where speed and accuracy in virus detection are critical for containment and response strategies.

## Results

### Design principle and structural characterizations

**Fig. 1:**
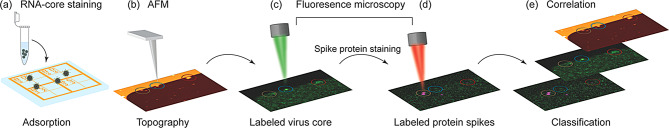
Experimental procedure: (**a**) Staining of the RNA-containing virus core and sample adsorption, (**b**) topography scanning followed by (**c**), (**d**), fluorescence microscopy imaging at two specific wavelengths (interrupted by the staining of the spike protein), (**e**) correlation of the three images

To unequivocally classify SARS-CoV-2, correlative experiments with fluorescence microscopy were performed as illustrated in Fig. 1.

In the first step, the RNA-containing core of SARS-CoV-2 was specifically stained, the virus particles were adsorbed on specifically designed glass substrates (a) and the topography was scanned with an AFM (b). The sample was then transferred to a fluorescence microscope and the same area was imaged at a wavelength that specifically excited the dye of the labelled core (c). The sample with the immobilized virus particles was then incubated to label the spike proteins on the virus and imaging was repeated with another specific wavelength (d). Finally, the three images were correlated with each other to classify the different species identified (e).

SARS-CoV-2 containing suspensions were isolated from cell cultures and inactivated with a 4% PFA solution. Besides glutaraldehyde and β-propiolactone, this is a commonly used chemical method for inactivating viruses by crosslinking proteins [42–44]. It has been reported that this procedure maintains the virus morphology and protein secondary structure of SARS-CoV-2 [2, 15, 22, 31]. Only at certain conditions changes in the morphology were observed [31]. 

### AFM topography of SARS-CoV-2

**Fig. 2:**
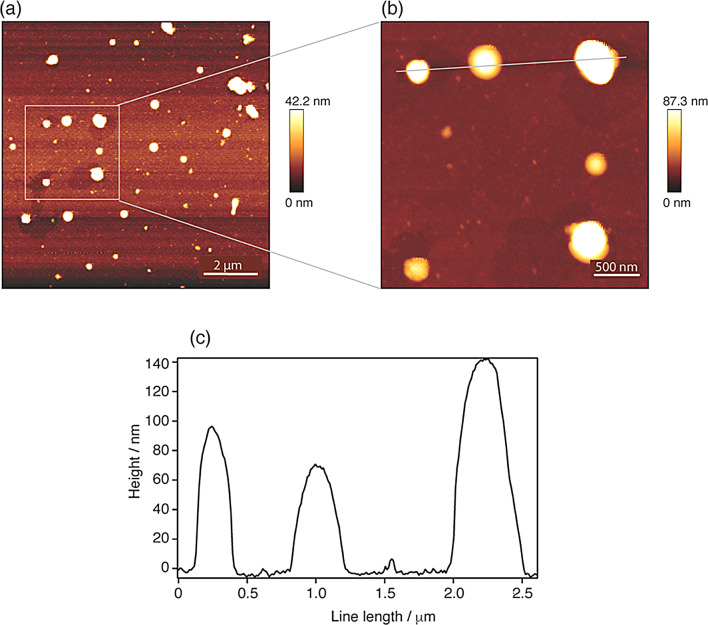
(**a**) Representative AFM topography of a PFA inactivated SARS-CoV-2 containing sample on a bare glass substrate, (**b**) detailed image of the area indicated by a white square in (**a**), (**c**) profile line across three individual particles in (**b**). Note that the color bars in the AFM images represent heights in the first standard deviation region, for better representation of smaller particles. For the absolute height the profiles are used

To exclude the possibility that the labeling of the core changes the height of the viruses, the topography of the non-modified viruses was first examined by drop cast the sample on bare glass cover slips. Figure 2a shows a typical AFM topography scan of the sample surface with spherical particles.

From the profile lines across three particles (detailed images in Fig. 2b and c), heights between 50 and 140 nm were determined. The topography image and the profile indicates that the highest structure is not a single particle, but an agglomeration of at least two particle (note the asymmetry of the rightmost profile), hence this structure would have beed excluded from the single particle experiments. Since bovine serum albumin is added for the cultivation process, cross-linking of protein residues in the sample during PFA fixation can lead to the formation of further particles, which could have a similar size and shape to the viruses, rendering a differentiation slightly more complex.

Following an established protocol [45], a cultured virus sample was labelled with SYBR gold, which specifically binds to the viral RNA. To ensure that identical areas were examined by AFM and fluorescence microscopy, glass substrates with fiducial markers were used. Prior to virus immobilization, the substrates were coated with a poly-L-lysine (PLL) layer which increased the adhesion of the virus particles to the substrate and thus the number of particles detected.

### Triple AFM-fluorescence correlation of SARS-CoV-2

**Fig. 3:**
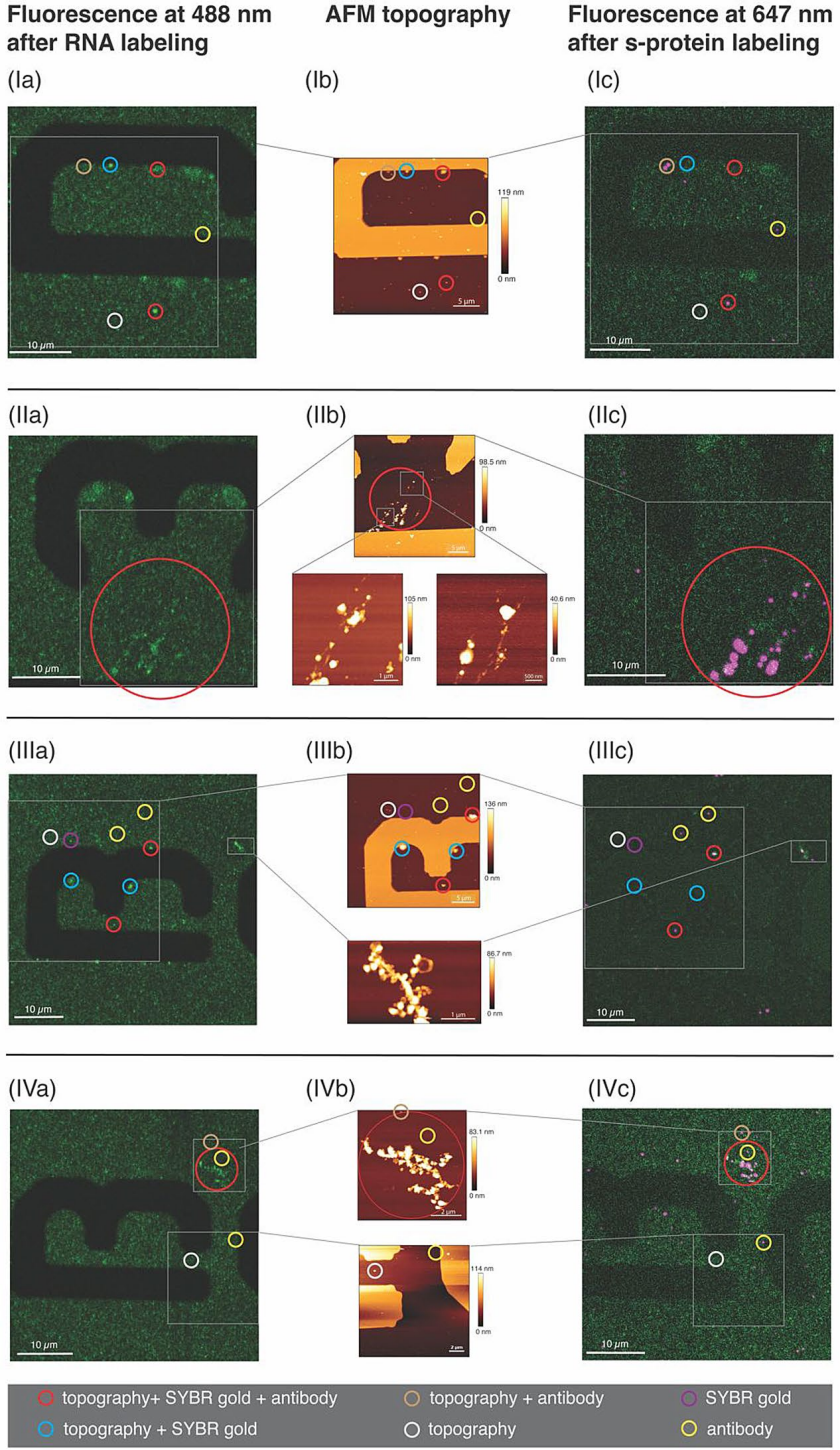
Triple AFM-fluorescence correlation of PFA-inactivated SARS-CoV-2 particles from four different areas (**I-IV**) on a PLL-coated glass substrate after SYBR-gold and subsequent antibody labeling. Center column (**I-IVb**): AFM topographies of the sample after SYBR gold labeling. Left column (**I-IVa**): Confocal fluorescence microscopy images upon 488 nm excitation of SYBR gold containing SARS-CoV-2. The grey squares indicate the areas of the AFM topographies in (**I-IVb**); Right column (**I-IVc**): Overlaid fluorescence microscopy images acquired after AFM imaging and s-protein immunolabelling upon 647 nm (Alexa Fluor 647 magenta) and 488 m excitation (SYBR gold, green). The grey squares indicate the areas of the AFM topographies in (**I-IVb**)

When using topographic imaging techniques for identification, it is crucial to have an independent proof which particles are viruses. If the sample contains cell debris, protein residues or particles with comparable height and morphology to the virus, a classification based on height alone is not always possible, due to the lack of biochemical information. One possibility to unambiguously classify a virus particle is the correlation of the AFM topography with conventional fluorescence microscopy images. Figure 3 shows the fluorescence and correlated AFM topography images of selected areas on the sample surface. More detailed images are given in the Supporting Information Figure S1.

Initially, the topographies were scanned and four different areas on the sample were selected (see Fig. 3I-IVb) where particles with the expected heights of SARS-CoV-2 virus (~ 50–100 nm) were found. In the next step, the sample was transferred to a fluorescence microscope and illuminated with 488 nm to excite the SYBR gold dye in the virus core. It is evident that most of the particles with a height above 50 nm in Fig. 3I-IVb appear as bright green spots in Fig. 3I-IVa which indicates the detection of SARS-CoV-2, i.e., the viral RNA. Finally, the sample was subjected to antibody labelling, whereby the spike (S)-protein was specifically labelled with an antibody attached to a red-emitting fluorescent dye and irradiated with 647 nm for fluorescence detection. It is important to mention that PFA-based inactivation does not affect the binding activity of the antibody to the virus [15, 42]. 

For better visibility, we combined the fluorescence images in Fig. 3I-IVc, here indicated as magenta spots, with those from Fig. 3I-IVa. The comparison shows that many of the bright magenta spots overlap well with the AFM images and with the green spots from the SYBR gold labelling experiment. In Fig. 3I-IV such events are marked with red circles for selected particles. Since the height profiles of these spots were between 60 and 100 nm, they can be clearly classified as intact SARS-CoV-2. Another class of particles showed topography, but a fluorescence signal was detected for only one of the two labels, as shown by the selections marked with blue circles in Fig. 3I, III and brown circles in Fig. 3I, IV, respectively. This observation indicates the presence of incomplete viral particles in the sample.

Selected fluorescent spots with no obvious topography are marked in purple (Fig. 3 III) and yellow (Fig. 3I, III, IV) and might correspond to unspecific labelling or labelling of viral fragments. The last class of particles was detected in the topography but did not show fluorescence in either of the experiments. Examples of those areas are highlighted with white circles in Fig. 3I, III, IV.

In summary, in the investigated sample areas, 41 particles with a height of 60–100 nm were detected in the AFM images, while for each particle both fluorescence labelling methods were positive. Interestingly, the correlation enables the assignment of specific structural properties of distinct particles that one experiment alone cannot provide. In the next step, the results were analysed in more detail by categorizing the detected particles by height and fluorescence event.

### Single-particle characterization of SARS-CoV-2 by combined morphological and fluorescence signatures

For this purpose, the respective particle height was determined via a line profile across each particle in the examined areas. Next, the particles were annotated as “positive” or “negative” according to the respective fluorescence information. Finally, the particles were classified and counted. Table 1 summarizes the results from the 199 evaluated particles/fluorescence events. 21 particles of those were agglomerates (height > 100 nm, see categories 1b, 2b, 4a).

Since the sample was prepared by incubation of an aliquot of the virus suspension, it was not possible to control agglomeration. This resulted in random partial aggregation and a clear correlation of fluorescence to a specific particle is impossible due to the diffraction limited imaging of the optical methods used (see Supporting Information Figure S2). Consequently, those particles were consequently excluded from further analysis. The last column in Table 1 therefore relates the percentage to the remaining 182 isolated particles.

**Fig. 4:**
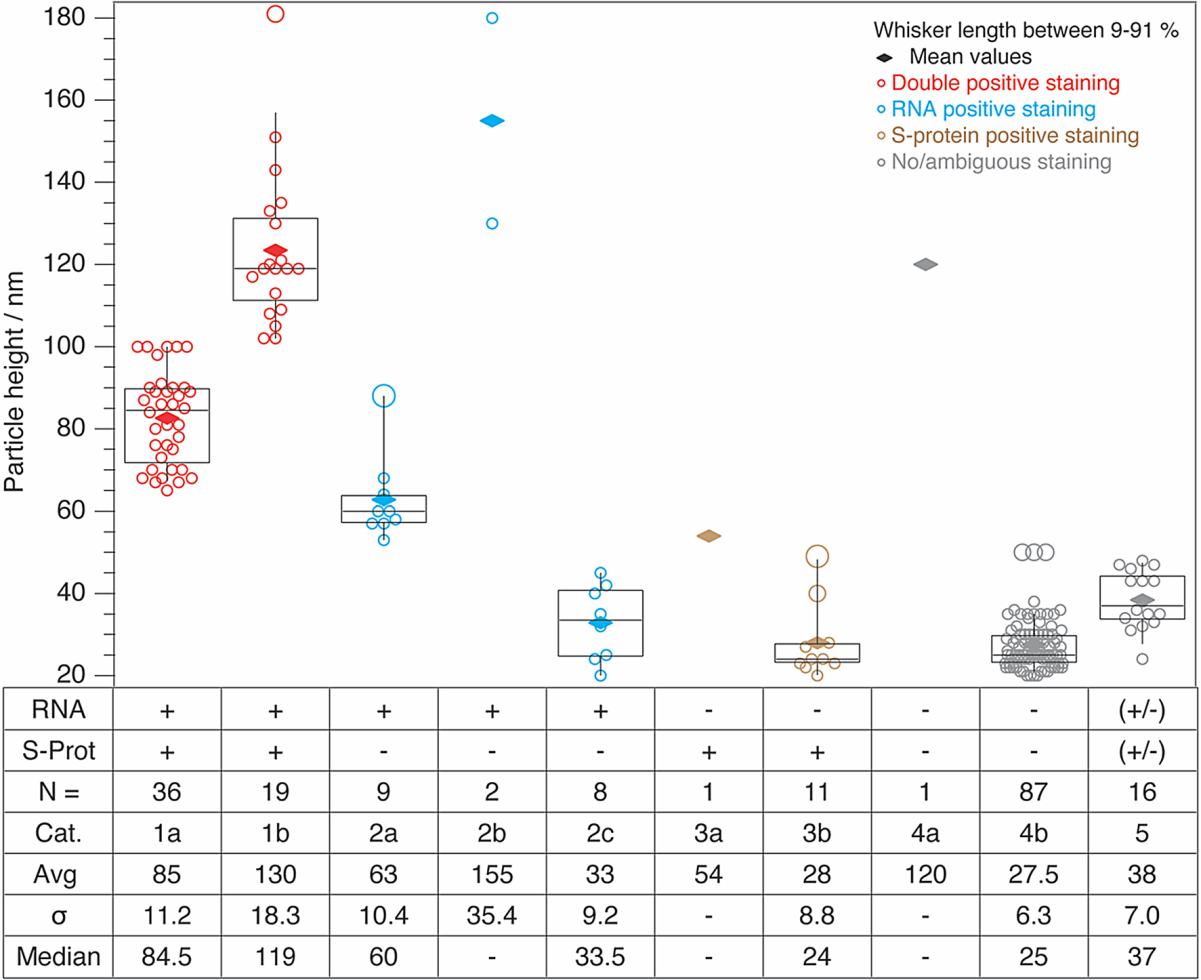
Box whisker plot of the data shown in Table 1. The boxes represent the quartiles and range from 25–75% of the particle heights. The medians are represented by black lines in the boxes and the mean by coloured diamonds. Data points for each category are indicated by circles using the same colour scheme as in Fig. 2. The x-axis annotation refers to Table 1 in addition with the mean, median and standard deviation; positive and negative staining events in the respective fluorescence images are indicated by + and -

20.3% of the particles investigated were positive for both RNA and spike protein (Table 1, category 1a, Fig. 4). As mentioned before, these particles can be unequivocally identified as SARS-CoV-2, albeit inactivated, with a height ranging from 60 to 100 nm. In agreement to other imaging methods used, our results also confirms that SARS-CoV-2 have a roughly uniform spherical structure. Consequently, it is reasonable that the height range determined in our present AFM topographies are equal to the actual diameter and thus agree nicely with the EM data mentioned before.

From the remaining ~ 80%, 9 particles (category 2a) had a slightly lower height, median 60 nm, than category 1a (48–90 nm, median 84.5 nm). Interestingly these particles were tested positive for RNA only, but negative for the S-protein. This would agree with recent observations that not all SARS-CoV-2 particles have protruding spikes [2, 22]. The AFM determined height agrees with the generally smaller height of such “spike-free” particles, however, as more specific information is missing, we presently cannot rule out RNA or DNA containing vesicles.

More than 50% of the particles were smaller than the proposed 60 nm as a minimum height criterion (categories 2c, 3b, 4b). These particles either showed no fluorescence at all or only in one condition and consequently would not be considered as potentially infectious. Interestingly, only one particle was identified as exclusively S-protein positive (category 3a). In this case, a virus-like particle lacking RNA could be assumed, but as only one such case appeared in the present study this is of course not fully conclusive. However, it has been shown earlier, that the height range of such hollow particles is in the range of 50–70 nm [46, 47].

The remaining three categories 5–7 did not show viable data for all three experiments and were excluded as virus particles. Among them, 16 particles (~ 9%, category 5) showed much weaker fluorescence intensities compared to the other categories and had heights below the defined threshold of viral height. Consequently, we consider those as too small for intact/complete viruses (albeit it could be still infectious material). The remaining 9 particles’ height (categories 6, 7) was below the noise level of the samples and obviously cannot be classified as viruses. However, as bright spots in one of the fluorescence images were detected, these were presumably SYBR gold and antibody residues, respectively.

Finally, it must be mentioned that incomplete labelling must also considered, whereby putative particles with the appropriate size were not detected as viral particles. However, no particles with fitting sizes were found, which is particularly obvious in Fig. 3. This indicates that the staining process as such is very efficient and specific, and the height alone is already a very specific parameter for an identification of active virus particles once the correlation experiments has been done once.

Using the height alone, only one false positive assignment would have been made in the present data set in the 10–90 percentile range. Compared to labelling methods, this is a surprisingly high specificity. Furthermore, from Fig. 4 it is apparent that there is almost no overlap in the particle sizes for the individual categories. In other words, each fluorescence condition can be assigned to a specific particle height.

In summary, the results show that all 60–100 nm high particles in category 1a are essentially complete viral particles, even RNA-free hollow particles and most S-protein-free particles can be distinguished via the height. This strongly indicates that in future experiments it will be possible to address height and shape/morphology determination by AFM for a pre-categorization of virions after separation from the cell culture and inactivation at a single particle level even without the need of labelling for a first assessment. Virus fragments, free RNA and S-protein residues could be excluded with this procedure. This renders height discrimination based on AFM a valuable tool at least for a fast pre-classification, as no special sample preparation is required. Furthermore, rather than only the height, AFM experiments can assess further parameters under the same experimental conditions, like the shape, friction, and even elasticity parameters etc. [48, 49] that would even further improve virion identification in a single image scan. It is, nevertheless, interesting that a quick height survey, provides already surprisingly precise possibility to distinguish even complete from incomplete virion material.

**Table 1:** Evaluation of the correlative AFM and fluorescence microscopy experiments of PFA-inactivated SARS-CoV-2 samples. 199 separate locations with at least one positive (fluorescence) result were detected. 21 particles > 100 nm were excluded as potential agglomerates, consequently 100% refers to 182 particles

Category	AFM height/ nm	RNA via SYBR gold staining	S-protein via antibody staining	Colour tag in Fig. 3	Count	Comment	%
1a	60–100	positive	positive	red	36	complete virus	20.3
1b	> 100	positive	positive	red	19	agglomerates (no clear assignment possible)	excl.
2a	48–90	positive	negative	blue	9	virus without S-protein or RNA containing vesicle	5.1
2b	> 100	positive	negative	blue	2	agglomerates	excl.
2c	20–47	positive	negative	blue	8	too small/virus fragment?	4.5
3a	54	negative	positive	brown	1	hollow particle/virus like particle	0.6
3b	20–50	negative	positive	brown	11	fragment with S-protein	6.2
4a	> 100	negative	negative	grey	1	not SARS-CoV-2 (and too big)	excl.
4b	20–50	negative	negative	grey	87	not SARS-CoV-2	49.1
5	30–45	doubtful	doubtful	grey	16	too small	9
6	no	negative	positive	yellow	7	virus fragment with S-protein or isolated S-protein	4
7	no	positive	negative	purple	2	small virus fragment with RNA or isolated RNA	1.1

## Materials and methods

### Virus propagation and characterization

SARS-CoV-2 variant SARS-CoV-2/hu/Germany/Jena-vi005587/2020 was isolated from a respiratory specimen of a patient from the Jena University Hospital (ethics approval no. 2018 − 1263) and titrated via standard plaque assay as previously described [50]. The virus strain was cultivated and propagated in Vero-76 cells cultivated in Dulbecco’s modified Eagle’s medium (DMEM) (Sigma-Aldrich, Taufkirchen, Germany) supplemented with 10% fetal bovine serum (FBS) (PAN-Biotech, Aidenbach, Germany) 1% Penicillin/Streptomycin (Biozym, Oldendorf, Germany). The determined sequence is available in the NCBI Genbank under the available accession number MW633323.

### SARS-CoV-2 inactivation

To analyse SARS-CoV-2 in laboratories outside BSL-3, we performed inactivation with paraformaldehyde (PFA). As an already established method of chemical inactivation, virus stocks were diluted 1:5 with 4% PFA in aqua bidest. and incubated for 30 min at 37 °C with 5% CO_2_ [51]. In detail, 400 µL 4% paraformaldehyde (PFA) solution was added to 100 µL virus suspension and incubated for 30 min at 37 °C to achieve complete inactivation. The mixture was vortexed and centrifuged at 14,000 rpm for 10 min. The supernatant was removed and 500 µL deionized H_2_O was added to rinse the virus sample followed by centrifugation at 14,000 rpm for 10 min. After the inactivation process and to eliminate medium-containing phenol red and salts, 100 µl of the inactivated supernatants were washed twice with aqua bidest. and finally resuspended in 100 µl of aqua bidest.

After the removal of the supernatant, the 10 µL of the virus suspension was deposited on a pre-cleaned (HNO_3_/H_2_O_2_ (3:1), 2.5 h) cover slip. After drying, the sample was used as is for the AFM topography measurements in Fig. 2.

### Concentration, purification, and staining of viral RNA with SYBR gold dye

The protocol was carried out adapted to the method published by Dent and Neuman [45]. At first, a fresh virus stock was prepared as mentioned above, yet in a specific serum-free virus propagation medium VP-SFM (Gibco/Thermo Fisher Scientific, Waltham, USA). After causing a sufficient cytopathic effect (CPE) in the Vero-76 cell layer, the virus-containing supernatant was filtered with a 0.45 μm sterile filter (Sarstedt AG & Co. KG, Nümbrecht, Germany) and centrifuged for 30 min, at 14,000 rpm, 4 °C. The pellet was subsequently resuspended in HEPES-saline (0.9% NaCl (w/v), 1mM HEPES, pH adjusted to 6.7) supplemented with one-tenth of the volume of the tracking dye 1x SYBR Gold Dye (Thermo Fisher Scientific, Waltham, USA). The solution was carefully added to a 30-40-50% sucrose gradient and centrifuged for 90 min, at 14,000 rpm, 4 °C without brakes. Each layer of the gradient was composed of 8 ml of the corresponding amount of sucrose in HEPES-saline. After centrifugation the viral RNA-containing layer was visualized with UV light, carefully extracted from the gradient, and centrifuged for 60 min, at 14,000 rpm, 4 °C. Finally, the pellet was resuspended in 100 µl HEPES-Saline.

### Immunofluorescence staining of SYBR gold labelled SARS-CoV-2 samples after AFM topography measurements

SYBR gold labelled SARS-CoV-2 covered slides were incubated with 5% bovine serum albumin (BSA) for 30 min at room temperature. Afterwards, the slides were incubated with the mouse SARS-CoV-2 spike antibody (1A9) (GeneTex; Irvine, USA, Cat#GTX632604 1:500 diluted in aqua bidest. for 90 min. After 2 washing steps with aqua bidest., the slides were further incubated with the secondary Alexa Fluor^®^ 647 AffiniPure Goat Anti-Mouse IgG (H + L) (Jackson Immuno Research Labs, West Grove, Cat#115-545-146 1:1000 in aqua bidest. for 1 h. After two more washing steps, the slides were used as is for the fluorescence microscopy measurements in Fig. 3a and c.

### AFM topography measurements of SYBR gold labelled SARS-CoV-2 samples

Patterned glass cover slips were pre-cleaned with HNO_3_/H_2_O_2_ 3:1 for 2. 5 h, thoroughly rinsed with deionized water and dried. 20 µL of a 0.0.1% poly-L-lysine (PLL) solution was incubated for 30 min, sucked off and the substrate was washed three times with deionized water. 5 µL of the SYBR gold labelled virus suspension was incubated on the substrate for 30 min, sucked off, rinsed twice with 5 µL water and dried. The sample was mounted on an inverted microscope equipped with an AFM (Nanowizard I, JPK-Bruker, Germany). For AFM imaging, we acquired data in tapping mode in air by using Budget Sensors TapAl-190G cantilevers. This provided stable imaging without lateral dragging and enabled robust three-way correlation with fluorescence channels. Imaging in liquid/buffer is in principle feasible provided reliable localization; however, absolute height depends on fixation and environmental conditions, so height ranges must be established for each fixation/inactivation procedure. For topography scanning in Fig. 2b, a 256 × 256 pixel resolution and Budget Sensors TapAl-190G cantilevers were used.

Manual pre-selection excluded objects with clear shoulders in line profiles or the amplitude-channel as evidence of more than 1 object. Because an AFM overestimates the true lateral width, due to tip–sample convolution, for the present assessment we quantified particles by height only; potential flattening of the virus on the surface does not affect our height–two-dye correlation.

The height assessment resulted in the exclusion of particles larger than 100 nm as agglomerates or stacked systems (see supporting information). This ensured the investigation of single particles. This is important, because stacked single particles might indeed fall into a different class in Fig. 4 when accidentally analyzed as one particle.”

### Fluorescence microscopy measurements labelled SARS-CoV-2 samples

Fluorescence microscopy images were acquired on an Abberior STEDYCON confocal microscope (Abberior Instruments, Göttingen, Germany) mounted on an Olympus IX83 microscope body (Olympus, Tokyo, Japan) equipped with a 100×/1.40 NA UPlanSApo Olympus objective lens. SYBR gold was excited with a 488 nm laser and Alexa Fluor 647 with a 640 nm laser. The laser power at the sample plane was 10 µW. Emitted photons were collected by the objective lens, descanned, passed through a 1.0 AU pinhole and recorded by avalanche photodiodes with 500–550 nm and 650–700 nm filters for 488 nm and 640 nm excitation respectively. Pixel size was 100 nm and pixel dwell time 10 µs.

## Discussion

The combination of AFM with targeted fluorescence labelling, presented in this study, provides a powerful and highly specific strategy for the structural and molecular characterization of SARS-CoV-2 at the single-particle level. The core advantage of this approach lies in its ability to correlate topographical data with molecular identity, overcoming a major limitation of conventional virus detection methods which often rely on either morphology or biomolecular labelling alone. Notably, our results confirm that the height range of 60–100 nm, determined via AFM, corresponds well with previously reported dimensions of intact SARS-CoV-2 particles obtained through cryo-electron microscopy and other imaging methods [2, 14, 23, 31]. This congruence reinforces the suitability of AFM as a label-independent, yet highly accurate, readout parameter for virus particle integrity.

The implementation of dual fluorescence labeling, targeting both the RNA core and S-protein, allowed us to clearly distinguish between complete and incomplete virions and potential staining artifacts. Importantly, particles displaying both RNA and S-protein labeling—category 1a in our classification—were consistently found within the established height range of 60–100 nm. These particles can therefore be considered intact, albeit inactivated, SARS-CoV-2 virions. Interestingly, particles positive for RNA alone (category 2a) but lacking S-protein labeling displayed slightly reduced height values, a finding consistent with prior observations that a subset of SARS-CoV-2 particles may naturally lack spike proteins on their surface [22, 23]. While their identity as virions cannot be conclusively established without further molecular confirmation, their presence highlights the inherent heterogeneity of virus populations and the importance of multimodal analysis. While we demonstrate two-colour specificity using SYBR nucleic acid staining and anti-spike immunolabeling, antibody dependence may delay application to newly emerging viruses. For enveloped virions, generic membrane dyes (e.g., DiO/DiI/DiD or FM-dyes) provide an antibody-independent second channel. To mitigate dye-aggregate artefacts, we recommend (i) dye-alone controls on the substrate, (ii) parallel detergent treatment to confirm membrane origin of signal, and (iii) co-registration with AFM particle topography. Incorporating a membrane dye maintains rapid turnaround while preserving the orthogonality of AFM-guided particle selection.

More than half of the evaluated particles fell below the height threshold of 60 nm or exhibited incomplete or no fluorescence signal. These were classified as virus fragments, protein aggregates, or free dye residues—particularly plausible given the presence of bovine serum albumin in the culture medium and the known limitations of diffraction-limited fluorescence microscopy. This finding further emphasizes the critical role of AFM height profiling in filtering out non-viral background signals, and in avoiding false positives which could compromise the specificity of purely fluorescence-based detection approaches.

It is particularly noteworthy that particle height alone, in combination with the established 60–100 nm threshold, was already a highly discriminative parameter. Among the 182 evaluable particles, only a single instance would have been misclassified using height alone. This striking specificity positions AFM as a promising tool for rapid pre-classification of viral particles—potentially even in label-free workflows. Beyond height, AFM also holds the capacity to measure additional mechanical and morphological parameters such as particle stiffness, friction, or adhesion properties [52]. These could, in future implementations, provide an even more nuanced fingerprint for virus detection and classification, especially in cases where labeling reagents are unavailable or compromised.

Limitations of our study include possible tip-induced deformation of soft viral envelopes, dehydration-related flattening. However, both effects would result in a systematic height offset based on the sample preparation and the applied AFM parameters. However, compared to the actual size spread of the viruses in the experiments such height changes are expected to be much smaller. Inactivation and labeling may alter antigen accessibility and fluorescence yield. We mitigated these issues by using low-force tapping and conservative particle pre-selection based on height/profile shape. Future liquid-mode force mapping can further reduce deformation while adding nanomechanical contrast.

The broader applicability of this approach lies in its versatility. By adapting the labeling strategy with virus-specific antibodies and nucleic acid dyes, the same methodological framework can be transferred to other RNA viruses of high pandemic potential, such as influenza viruses, arboviruses (e.g., Dengue, Zika), and other emerging coronaviruses. Given the early-stage unpredictability and high transmissibility of such pathogens, rapid and unambiguous particle identification becomes a cornerstone for effective outbreak response. The ability to structurally and molecularly classify virions without the need for extensive preparation or culture steps offers a distinct advantage in clinical and field settings.

Taken together, the combined AFM-fluorescence approach demonstrated here represents a significant step forward in nanoscale virus identification, offering high specificity, structural resolution, and adaptability. In future pandemics, or in the surveillance of known high-risk viruses, such a platform could serve as a powerful diagnostic tool, bridging the gap between ultrastructural characterization and molecular specificity in a single, correlative imaging assay.

## Conclusions

For virus characterization on the single particle level, it is often desirable to achieve a quick pre-selection of potential candidates, for example by AFM morphology. A direct a priori identification on morphology alone, however, is not possible without further structural information particularly when novel virus strains are involved. This limitation is especially relevant in clinical settings, where the rapid emergence of new viral variants requires precise differentiation to guide appropriate diagnostic and therapeutic measures. In the present study, a triple correlative approach involving conventional fluorescence microscopy as a reference was used to identify and classify viral material. For this, RNA-containing SARS-CoV-2 was used as a model specimen. The core and envelope of the virus were specifically labeled separately with dyes and antibodies, respectively, and excited with distinct wavelengths. The use of specially patterned glass substrates facilitated the location of individual virus particles with the different microscopy techniques. The subsequent fluorescence-height correlation clearly demonstrated that the height (size) of intact SARS-CoV-2 varied between 60 and 100 nm with a mean value of 85.4 nm, a median value of 86 nm and a standard deviation of 13 nm. In the experiments, only complete virions showed positive twofold fluorescence events, clearly limiting the height variability of the viruses to a narrow range. This allowed even RNA- and S-protein free particles to be distinguished via height measurements, both of which showed signals in only one of the two fluorescence experiments. The correlative data also indicated further viral material like agglomerates (height > 100 nm) and fragments, exposed RNA and s-protein residues (height < 50 nm).

In essence, the results highlight the increased specificity resulting from the correlation of AFM and fluorescence images, as well as the possibility of targeting height determination as a suitable tool for pre-selection of viral particles. The presented approach of correlating scanning probe and conventional fluorescence microscopy can be applied to any other viral sample for which the antibody is known. It can further contribute to a targeted pre-selection and pre-characterization of viral sample material and can serve as a starting point for further, more detailed analysis. While AFM is currently not established in routine diagnostics, its phenotypic readouts are particularly valuable in early outbreak settings before sequence-specific assays are available, and for applications such as assessing particle integrity/heterogeneity or probing viral tropism. Accordingly, we envisage deployment as a same-day adjunct in specialized reference laboratories with appropriate BSL-2/3 infrastructure, rather than point-of-care use.

## Supplementary Information

Below is the link to the electronic supplementary material.

Supplementary Material 1

## Data Availability

The datasets generated and/or analyzed during the current study are available from the corresponding author on reasonable request.
