# Supplementary material for: Atomic force microscopy-based topographical imaging of SARS-CoV-2 as part of a tripartite strategy for RNA virus characterization
Source: J Transl Med. 2025 Dec 9;24:233. doi: 10.1186/s12967-025-07490-3 (PMC12903297; doi:10.1186/s12967-025-07490-3)
Supplement: Supplementary file 1 — Supplementary Material 1 [file 12967_2025_7490_MOESM1_ESM.docx]

Supplementary Information

**Atomic Force Microscopy-Based Topographical Imaging of SARS-CoV-2 as Part of a Tripartite Strategy for RNA Virus Identification**

T. Deckert-Gaudig^1,2^, Xiaobin Yao^1,2^, Erwan Darussalam^1,2^, Franziska Hornung,^3^ Pablo Carravilla^4,5,6^, Ziliang Zhao^4,5^, Kourosh Rezaei, Christian Eggeling^4,5,7^, Stefanie Deinhardt-Emmer^3^*, Volker Deckert^1,2^*

Due to the size, the image appear on the following separate pages

**S1.** Details of the AFM-fluorescence correlation experiments of PFA-inactivated SARS-CoV-2 (**Fig. 3** in the manuscript). **Ib, IIIb, IVb**: AFM topographies after SYBR gold labeling. The grey lines show the profile lines drawn across the particles to determine their height. The grey squares indicate where sample areas were scanned in more detail; **Ia, IIIa, IVa**: Fluorescence microscopy images sat excitation with 488 nm. Green spots point to SYBR gold containing SARS-CoV-2. The grey squares indicate the areas of the AFM topographies in **Ib, IIIb, IVb**. **Ic, IIIc, IVc**: Fluorescence microscopy images at excitation with 647 nm after additional s-protein antibody-dye labeling. To improve the visibility, the images were overlaid with the images **Ia, IIIa, IVa.** Magenta spots point to antibody labeled SARS-CoV-2. The grey squares indicate the areas of the AFM topographies in **Ib, IIIb, IVb**. The colored circles highlight particles of specific interest discussed in the manuscript.


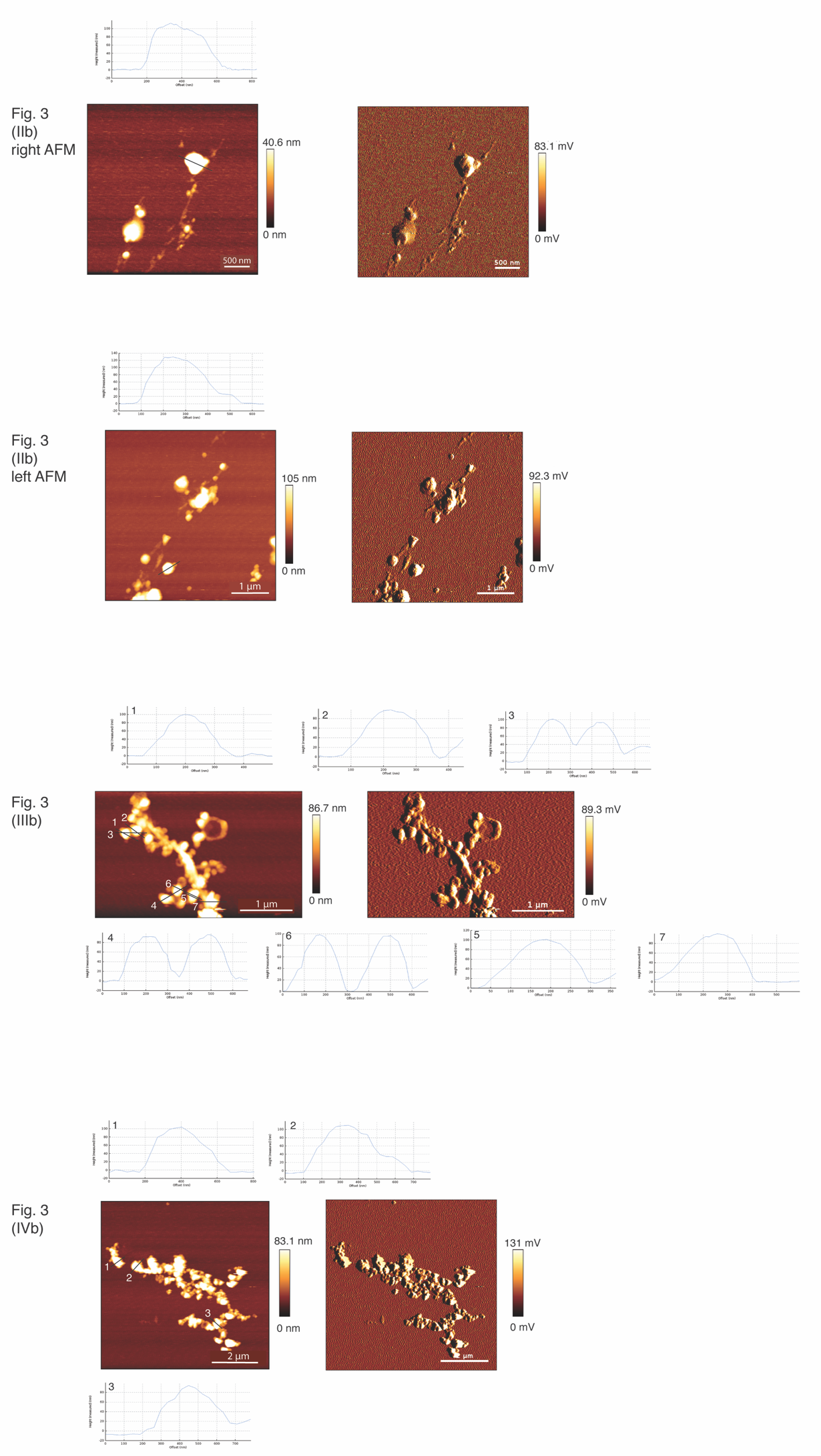


**S2.** Height determination of the agglomerates imaged in the AFM-fluorescence correlation experiments of PFA-inactivated SARS-CoV-2 ( **Fig. 3** in the manuscript). The grey lines show the profile lines drawn across the particles. The respective figures to the right show the amplitude (often called error-signal or feedback signal) channel. That often helps to recognize potential shoulders or double particles.
